# Rapid prediction of winter wheat yield and nitrogen use efficiency using consumer-grade unmanned aerial vehicles multispectral imagery

**DOI:** 10.3389/fpls.2022.1032170

**Published:** 2022-10-24

**Authors:** Jikai Liu, Yongji Zhu, Xinyu Tao, Xiaofang Chen, Xinwei Li

**Affiliations:** ^1^ College of Resource and Environment, Anhui Science and Technology University, Fengyang, China; ^2^ Anhui Province Agricultural Waste Fertilizer Utilization and Cultivated Land Quality Improvement Engineering Research Center, Anhui Science and Technology University, Fengyang, China

**Keywords:** DJI Phantom 4 Multispectral (P4M) camera, grain yield, vegetation indices (VIs), winter wheat, nitrogen use efficiency (NUE)

## Abstract

Rapid and accurate assessment of yield and nitrogen use efficiency (NUE) is essential for growth monitoring, efficient utilization of fertilizer and precision management. This study explored the potential of a consumer-grade DJI Phantom 4 Multispectral (P4M) camera for yield or NUE assessment in winter wheat by using the universal vegetation indices independent of growth period. Three vegetation indices having a strong correlation with yield or NUE during the entire growth season were determined through Pearson’s correlational analysis, while multiple linear regression (MLR), stepwise MLR (SMLR), and partial least-squares regression (PLSR) methods based on the aforementioned vegetation indices were adopted during different growth periods. The cumulative results showed that the reciprocal ratio vegetation index (repRVI) had a high potential for yield assessment throughout the growing season, and the late grain-filling stage was deemed as the optimal single stage with R^2^, root mean square error (RMSE), and mean absolute error (MAE) of 0.85, 793.96 kg/ha, and 656.31 kg/ha, respectively. MERIS terrestrial chlorophyll index (MTCI) performed better in the vegetative period and provided the best prediction results for the N partial factor productivity (NPFP) at the jointing stage, with R^2^, RMSE, and MAE of 0.65, 10.53 kg yield/kg N, and 8.90 kg yield/kg N, respectively. At the same time, the modified normalized difference blue index (mNDblue) was more accurate during the reproductive period, providing the best accuracy for agronomical NUE (aNUE) assessment at the late grain-filling stage, with R^2^, RMSE, and MAE of 0.61, 7.48 kg yield/kg N, and 6.05 kg yield/kg N, respectively. Furthermore, the findings indicated that model accuracy cannot be improved by increasing the number of input features. Overall, these results indicate that the consumer-grade P4M camera is suitable for early and efficient monitoring of important crop traits, providing a cost-effective choice for the development of the precision agricultural system.

## Introduction

Wheat (*Triticum aestivum L.*) is the most crucial global staple food that constitutes 20% of the required calories and proteins for humans (Curtis and Halford, 2014). Since the “Green Revolution” of the 1950s, wheat yield has increased significantly with the application of nitrogen fertilizer (Nguyen et al., 2019). To increase wheat yield sustainably, agricultural systems worldwide have been extensively applying nitrogen fertilizers (Han et al., 2015; Ali et al., 2018). However, accumulating evidence suggests that the goal of increasing wheat yield through nitrogen application rate has reached a bottleneck (Fischer et al., 2009; Curtis and Halford, 2014). Meantime, the continuous excessive application of nitrogen fertilizer leads to an increase of agricultural production cost and irreversible environmental pollution (Sharma and Bali, 2018). Hence, current research is focused on how to maximize nitrogen use efficiency (NUE) while maintaining a reasonable N fertilizer application rate, so as to achieve the ultimate goal of increasing wheat yields (Good et al., 2004; Han et al., 2015; Ali et al., 2018; Nguyen and Surya, 2018).

Obvious genotypic differences exist in the yield and NUE, and complex polygenic traits are influenced by the genotype, management practice, and environment (Curtis and Halford, 2014; Han et al., 2015; Sharma and Bali, 2018). Researchers need to systematically examine the level of variation in different varieties with different N gradients and environments (Monostori et al., 2017). The assessment of yield and NUE traits of different varieties under natural conditions in the field is typically performed at the time of crop maturity by manually operating simple machines and through complex chemical analyses performed in laboratories. These processes are laborious, inefficient, and destructive. Moreover, the inefficiency of phenotype data acquisition capacity restricts the high-throughput development of crop genetic improvement (Prey et al., 2020; Jin et al., 2021). Thus, low-cost, robust, high-throughput phenotype acquisition platforms and technologies are highly warranted.

Compared with satellites and ground-based proximal sensors, a high-throughput phenotyping platform with unmanned aerial vehicles (UAVs) is an economical, practical, efficient, and non-destructive solution to conveniently capture spatial resolution images on centimeter- or millimeter-scale without the constraints of weather conditions (Lin, 2015; Yang et al., 2017; Hassler and Baysal-Gurel, 2019). This approach ensures data collection at critical crop growth stages, facilitating the efficient management or monitoring of crops.

Spectral information from UAVs is mainly used in the form of vegetation indices (VIs) (Araus and Cairns, 2014; Svensgaard et al., 2019; Guo et al., 2021). VIs represent mathematical transformation of reflectance of two or more bands to characterize the canopy spectral characteristics of crops (Yang and Guo, 2008; Qiu et al., 2018), and it is the simplest, most effective, and most widely studied method for the estimation of crop parameters (Feng et al., 2021). To obtain the ideal crop yield prediction accuracy, researchers have used the UAVs to explore the combination of VIs and stages that suits their respective research needs. For instance, Zhou et al. (2017) predicted rice yield by using UAVs-based RGB and multispectral imagery and demonstrated that airborne RGB and multispectral VIs could be used as reliable platforms for crop growth and yield estimation. Zhu et al. (2018) also used the UAVs equipped with a multispectral camera to acquire images of wheat at different growth stages and assessed the yield by using nine VIs. The analysis results showed that the most effective estimation model was presented from the heading to the filling stage, and the optimal vegetation index was an enhanced vegetation index without a blue band (EVI2). Fu et al. (2020) used a multispectral camera to obtain canopy images of wheat at the critical growth stages and predicted wheat yield by machine learning methods. The results revealed that the vegetation indices at the jointing, flowering, and filling stages showed reasonable fit efforts with the yield. Normalized difference vegetation index (NDVI) at the jointing stage, normalized difference red-edge index (NDRE) at the flowering stage, and canopy chlorophyll content index (CCCI) at the filling stage showed the best yield estimation. The study by Shafiee et al. (2021), on the other hand, concluded that NDVI for wheat yield was the strongest predictor, while the addition of MERIS terrestrial chlorophyll index (MTCI) in the pre-filling period could improve the predictive power of yield models. Wan et al. (2020) developed the best rice yield prediction model based on NDVI, normalized difference yellowness index (NDYI), canopy height, and canopy cover by using a UAVs high-throughput platform equipped with RGB and multispectral cameras. Moreover, the initial heading stage was considered the best stage for yield prediction. Results of previous studies suggest that the best predictive VIs and stage for yield modeling generally differ across the growth season. In other words, such yield estimation models can be applied at specific time points, and they show limited extrapolation capability under any other growth stage during the growing season. This point undoubtedly raises the threshold for spectral vegetation indices in applied production. Practitioners without specialized remote sensing knowledge prefer using a single vegetation index during multiple stages of the growing season.

Unlike yield prediction, the NUE traits have been less frequently evaluated by UAVs. Yang et al. (2020) evaluated the NUE of wheat varieties by using a UAV_mounted multispectral camera. The results revealed that the NDRE had a high consistency and accuracy for NUE, particularly in the mid to late-grain filling stage. In addition, the nitrogen dynamics time-series curves of the two rice populations were captured by UAVs’ multispectral imagery, which were used to identify an available spectral index (NDRE) and a high NUE variety (Liang et al., 2021). Therefore, more studies are needed to explore the potential of UAVs imagery in NUE prediction.

With the proliferation of the UAVs and sensor markets, increasing numbers of consumer-grade UAVs are being used for agricultural remote sensing research, and their use is being promoted by actual agricultural managers (Gallardo-Salazar and Pompa-García, 2020; Lu et al., 2020; Di Gennaro et al., 2022). The advent of the DJI Phantom 4 Multispectral (P4M) camera (SZ DJI Technology Co., Shenzhen, China) brings multispectral sensors into the consumer-grade category. However, there are few studies have evaluated cereal yield and NUE by using P4M multispectral imagery.

In this study, we used a P4M camera to develop rapid prediction models of yield and NUE traits that can be applied to different growth stages. In summary, we aimed to (1) determin the optimal VIs that can be applied to multiple periods within a growing season for yield and NUE traits assessment; (2) compare the performance of several linear regression (LR) models based on optimal VIs at different growth periods, and (3) validate the potential of the P4M camera for wheat grain yield or NUE prediction of precision agricultural systems. Based on a literature review and discussion of the current methodology, we present our research findings and discuss the optimal screening VIs and estimation models suitable for wheat trait monitoring during multiple growth periods. Our study can provide an efficient, convenient, and reliable high-throughput phenotype selection method to construct intelligent agricultural systems.

## Materials and methods

### Design of field experiment

**Figure 1:**
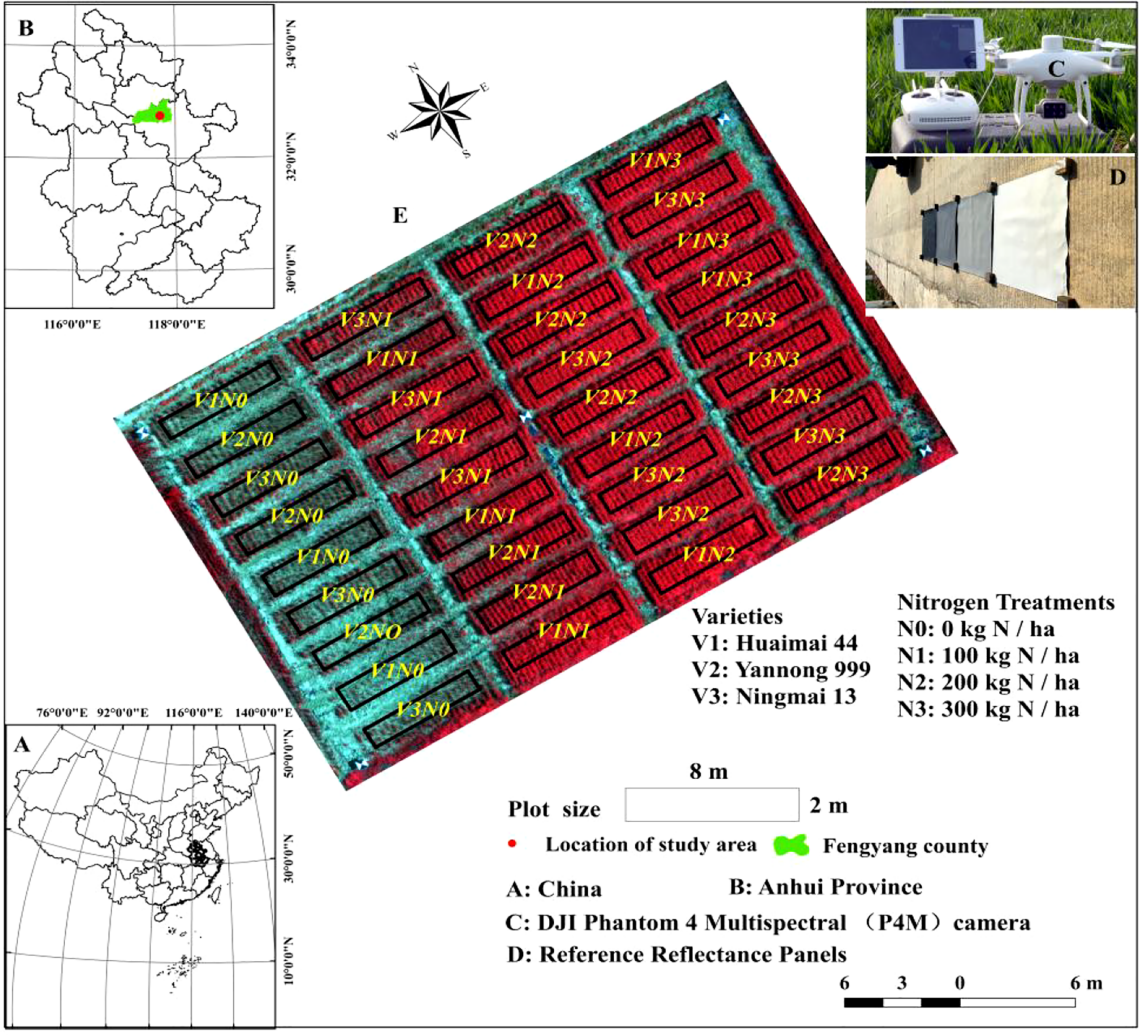
Experimental location **(A, B)**, plot design **(E)**, P4M platform **(C)**, and calibration panels **(D)** in the present study.

The experimental area was set up in Xiaogang Village, Anhui Province, China, located on the bank of Huai River. Typical warm temperate semi-humid continental monsoon climate of Xiaogang Village is 15.4°C annual average temperature with mean annual precipitation of 1236.2 mm, and summer maize and winter wheat are the major crops grown in this region. The experiment was conducted during the 2020–2021 wheat season, and was laid out using a split-plot design with three replicates, with nitrogen fertilizer levels assigned to the primary plots, whereas the winter wheat varieties were set up in subplots. There were four nitrogen fertilizer levels (N0 = 0, N1 = 100, N2 = 200, and N3 = 300 kg/ha) and three wheat varieties (V1: Huaimai 44, V2: Yannong 999, and V3: Ningmai 13). The three winter wheat varieties are newly released in Huang-Huai-Hai areas of China, and have the potential of high yield and stability. Black plastic films with 4 mm thickness were deposited between primary plots with different nitrogen fertilizer gradients to prevent water and fertilizer diffusion. Nine subplots of the primary plots were designed with 50-cm spacing. The whole study area comprised 36 wheat plots, with an area of 16 m^2^ (2 m×8 m) for each plot (**Figure 1**). Winter wheat seeds were sown on November 7, 2020, following rice harvest, and the rice stubble was treated in time, with a row spacing of 30 cm and artificial drilling. Irrigation was then performed to maintain the soil moisture content. In late February 2021, winter wheat enters the turning green and rising period. Then, in late April, it enters the flowering stage. In May, the winter wheat enters the grain-filling stage, and hence, the harvest was performed on June 3, 2021. Nitrogen fertilizers were applied at 60% and 40% during the sowing and jointing stages, respectively. Phosphate (P = 90 kg/ha) and potassium (K = 135 kg/ha) fertilizers were applied as basal fertilizers prior to sowing. For field management, local high-yield cultivation methods and pest and disease control strategies were adopted. Fortunately, the weather conditions were ideal throughout the winter wheat growing season, and no meteorological disasters such as drought or waterlogging occurred during this period.

### UAVs multispectral image acquisition

The DJI P4M camera was used for multispectral imagery acquisition; it is the latest consumer-grade quad-rotor multispectral imaging system that can be used for agricultural applications launched by DJI. It consists of 5 monochrome sensors of 12.08 megapixels and is configured on a 3-axis gimbal to obtain clear and stable images. In addition, the camera is equipped with a real-time kinematic (RTK) system to obtain images with centimeter-level positioning accuracy. **Table 1** describes the technical specifications of the camera.

**Table 1:** DJI P4M camera technical specifications.

Parameters	Values
Sensor	1/2.9 inch CMOS(5)
Pixel resolution(px*px)	1600*1300
Acquisition mode	snapshot
Optics	f/2.20
Focal length(mm)	5.74
FOV(°)	62.7
Battery life(minutes)	27
RTK accuracy(m)	vertical: ± 0.1; horizontal: ± 0.1
Photo format	.tiff
Bands set	Blue:450 nm ± 16 nm
	Green:560 nm ± 16 nm
	Red:650 nm ± 16 nm
	Red Edge:730 nm ± 16 nm
	NIR:840 nm ± 26 nm

The UAVs campaigns were conducted in clear, cloudless, and calm weather conditions between 11:00 and 13:00 local time. We employed the software DJI GS PRO software (https://www.dji.com/cn/ground-station-pro/) to pre-plan the routes and examine the aerial photography performance in real-time during the flight. Multispectral images were acquired from 30 m above the ground level, with a flight speed of 2.0 m/s, under automatic exposure mode, and 90% and 85% of overlap in the flight and side directions, respectively. A series of 6 campaigns with identical flight plans were developed using the P4M camera (**Table 2**). The photos were saved in the.tiff format, and 785 images were captured per flight.

**Table 2:** Flight details for the entire growth season.

Date	Growth stages	Heights(m)	Spatial resolution(cm*cm)
03/14/21	Jointing(J)	30	1.64*1.44
04/08/21	Booting(B)		1.85*1.54
04/18/21	Heading(H)		1.74*1.44
04/29/21	Late flowering(LF)		1.74*1.45
05/09/21	Initial grain-filling(IGF)		1.74*1.46
05/24/21	Late grain-filling(LGF)		1.74*1.47

### Ground hyperspectral data acquisition

Spectral measurements of the winter wheat canopy were conducted using the ASD FieldSpec HandHeld2 (ASD HH2) portable spectrometer (Analytical Spectral Devices, Boulder, Colorado, USA). The spectroradiometer can take continuous spectrum measurements in the 325–1075 nm wavelength range, with a spectral resolution of <3.0 nm at 700 nm, wavelength accuracy of 1 nm, and a view angle of 25°C. The measurements were performed under stable sunlight conditions before or after the drone flight, and radiation correction was performed with a standard whiteboard before measurements. Three representative uniform areas were selected for the measurement of each plot. The detector was downward, while the vertical distance was approximately 50 cm above the canopy. All spectra collected from the same plot were averaged to represent the mean reflectance of this plot. Considering the lack of spectral response functions of the P4M camera, we compared and analyzed the differences between the reflectance of the P4M bands and the field-measured mean spectral measurement at the plot scale to illustrate the reliability of the P4M multispectral camera.

### UAVs multispectral image preprocessing

Preprocessing is fundamental for ensuring better image quality and consistency in subsequent analyses, and it mainly includes orthophoto image generation, radiometric calibration, and geometric correction. The original multispectral photos (.tiff format) were evaluated to exclude images with any noticeable distortion. To generate orthophoto images, we used DJI Terra software (https://www.dji.com/cn/downloads/softwares/dji-terra), which ensures the extraction of accurate quantitative information by performing positional error registration and radiation distortion correction from exposure, vignetting, file format, and spectral sensitivity. Then, the 5 single-band orthophoto images were merged into a multispectral file (.tiff) by using ENVI software (Exelis Visual Information Solutions, Boulder, Colorado, USA). The unified coordinate system used was WGS_84 UTM 50N. Following preprocessing, multispectral images of the experimental field were obtained, and the spatial resolution was resampled to 1.5 cm.

We applied the empirical line method (ELM) to each band based on four reference panels with known reflectance values. Di Gennaro et al. (2022) demonstrated the effectiveness of the ELM model for radiometric correction of the P4M camera. To eliminate the boundary effect, an area of approximately 0.2 m × 0.2 m was selected in the central part of the reference panels, and the average value was extracted and used as the DN value of the reference panels. The DN values were further transformed into reflectance values by using the following equation:


(1) $$R_{\left(i,j\right)}=DN_{\left(i,j\right)}*a_{i}\;,\;i\in \left[1,5\right],\;j\in \left[1,4\right]$$

where *R*_(*i*,*j*)_ and *DN*_(*i*,*j*) _ are the reflectance and DN values of the reference panel *j* in band *i*, respectively, and *a*_*i*_ is the slope coefficient of ELM.

### Removal of the soil background

During the early growth period of winter wheat (i.e., at the tillering and jointing stages), the plants were short, and the soil background occupied a major proportion in the field of view, which led to an underestimation of the VI value. Zhu reported that the impact of soil background on early winter wheat yield estimation was remarkable (Zhu et al., 2018). In addition, plants in the non-fertilized (N0) area had less tillering and weak growth, and the vegetation coverage was low throughout the growing season. Referring to a previous study (Jay et al., 2019), we selected the visible atmospherically resistant index (VARI) and adopted the threshold method to remove the soil background. The winter wheat accuracy evaluation results after background removal based on the VARI threshold were presented in **Supplementary Table S1**.

### Calculation of vegetation indices

The reflectance of the 5 bands was extracted from the background-removed multispectral images, and nine vegetation indices, which have been widely used to assess crop yield and biochemical parameters, were calculated. Among these indices, MTCI used the red-edge band, which effectively weakened the reflectance changes caused by leaf orientation and specular reflection and had a positive effect on accuracy of the crop physiological parameter prediction (Dash and Curran, 2004). Modified normalized difference blue index (mNDblue) could effectively reduce the radiation error caused by the soil background and illumination changes, and it was insensitive to the canopy structure (Jay et al., 2017). Normalized green, red difference index (NGBDI) is calculated by bands in the visible light range, which is sensitive to the reproductive growth of crops, and it showed better for rice grain yield prediction than VARI (Wan et al., 2020). In another study, this index has been referred to as NDYI (John and Dan, 2016). Based on the ratio vegetation index (RVI), the present study proposes the reciprocal ratio vegetation index (repRVI), which was calculated by dividing the NIR band by the red band. The vegetation indices are detailed in **Table 3**.

**Table 3:** Multispectral vegetation indices used in this study.

VIs name	Algorithm formula	Reference
Visible atmospherically resistant index (VARI)	(R_green_−R_red_)/(R_green_+R_red_−R_blue_)	Gitelson et al., 2003a
Normalized green, red difference index (NGBDI)	(R_green_−R_blue_)/(_green_+R_blue_)	Hunt et al., 2005
Normalized difference vegetation index (NDVI)	(R_NIR_−R_red_)/(_NIR_+R_red_)	Rouse et al., 1974
Normalized difference red edge index (NDRE)	(R_NIR_−R_rededge_)/(_NIR_+R_rededge_)	Barnes et al., 2000
Green normalized difference vegetation index (GNDVI)	(R_NIR_−R_green_)/(_NIR_+R_green_)	Gitelson et al., 1996
Red edge chlorophyll index (CIrededge)	R_NIR_/R_rededge_−1	Gitelson et al., 2003b
MERIS terrestrial chlorophyll index (MTCI)	(R_NIR_−R_rededge_)/(R_rededge_−R_red_)	Dash and Curran, 2004
Modified normalized difference blue index (mNDblue)	(R_blue_−R_rededge_)/(R_NIR_+R_blue_)	Jay et al., 2017
Reciprocal ratio vegetation index (repRVI)	R_red_/R_NIR_	Jordan, 1969

repRVI is in a reciprocal relationship to RVI (Jordan, 1969).

### Agronomic data acquisition and preprocessing

At the physiological maturity stage of winter wheat, three representative and uniform 0.5-m double-row areas in each plot were selected for sampling. The harvested ears were transferred to the laboratory and sun-dried until the weight remained unchanged. The average yield of the 3 sampling sub-plots served as the final yield of the plot, and this yield was uniforml**y** converted to kg/ha.

Several definitions have been developed for NUE, and most of these definitions are based on grain yield, meaning the input-output ratio of nitrogen fertilizers (Moll et al., 1982; Good et al., 2004; Hawkesford, 2017). Agronomical NUE (aNUE) is calculated based on the grain yield under N application when compared with that under the 0 level, and it was used to assess the utilization efficiency of the fertilizer applied on top of the residual N in the soil, In addition, the N partial factor productivity (NPFP) is adjusted for the grain yield with the direct application of the N supply under each treatment (Wan et al., 2020). Both indicators emphasized the nitrogen fertilizer input-output ratio, which indicated the ability of the crop to efficiently use the applied nitrogen fertilizer to increase grain yield. aNUE and NPFP were calculated according to the formulas (2) and (3), respectively, which are as follows (Kefauver et al., 2017; Wan et al., 2020)


(2) $$aNUE=\frac{GY_{Ni}-GY_{N0}}{Ni}\;\;\;i\in \left[1,3\right]$$

(3) $$NPFP=\frac{GY_{Ni}}{Ni}\;\;\;i\in \left[1,3\right]$$

where *GY*_*Ni*_  and *GY*_*N*0_ represent the grain yield of the plot at the *i* (*i≠0*) level and 0 levels, respectively; and *Ni*  represents the N supply at the *i* (*i≠0*) level.

Variance analysis was implemented to describe the differences among the different N levels and varieties in terms of grain yield and NUE by using Wilcoxon rank sum and signed rank tests in RStudio (version 1.4.1106) (https://www.rstudio.com/) with R version 4.04 (https://www.r-project.org/).

### Model development and performance assessment

Linear regression (LR) is a simple model that incorporates the concept of naive machine learning modeling and serves as the basis for highly complicated linear models. The least-square method based on the minimization of mean square error is the basic method employed for solving the LR model. Multiple LR (MLR) model involves two or more independent variables and considers the comprehensive effect of multiple independent variables on the dependent variable. In the MLR model, multiple correlations among variables affect the estimation of parameters, thereby decreasing the estimation accuracy. Therefore, other methods are preferred to eliminate multicollinearity (Fu et al., 2020). Stepwise MLR (SMLR) is a modeling method that eliminates covariance by removing unnecessary independent variables through AIC value minimization iterations and selecting significant independent variables to obtain the optimal regression model. The SMLR model has simple logic and clear physical meaning of the independent variables, indicating that it is an interpretable machine learning model (Yu et al., 2016; Han et al., 2019; Zhang et al., 2022). Partial least-squares regression (PLSR) is one of the widely used machine learning methods that combines the basic functions of MLR, canonical correlation analysis, and principal component analysis. This method can avoid the non-normal distribution of data, eliminate the multi-linear relationship between independent variables, and maintain the relationship between independent variables and factors. PLSR has demonstrated satisfactory performance in agricultural remote sensing research (Fu et al., 2014; Kasim et al., 2017; Shu et al., 2021). Considering the limited number of samples in the current study, complex machine learning algorithms such as random forest (RF), support vector machine (SVM), and neural network (NN), which are recommended for processing high-dimensional features, were not applied in this study.

**Figure 2:**
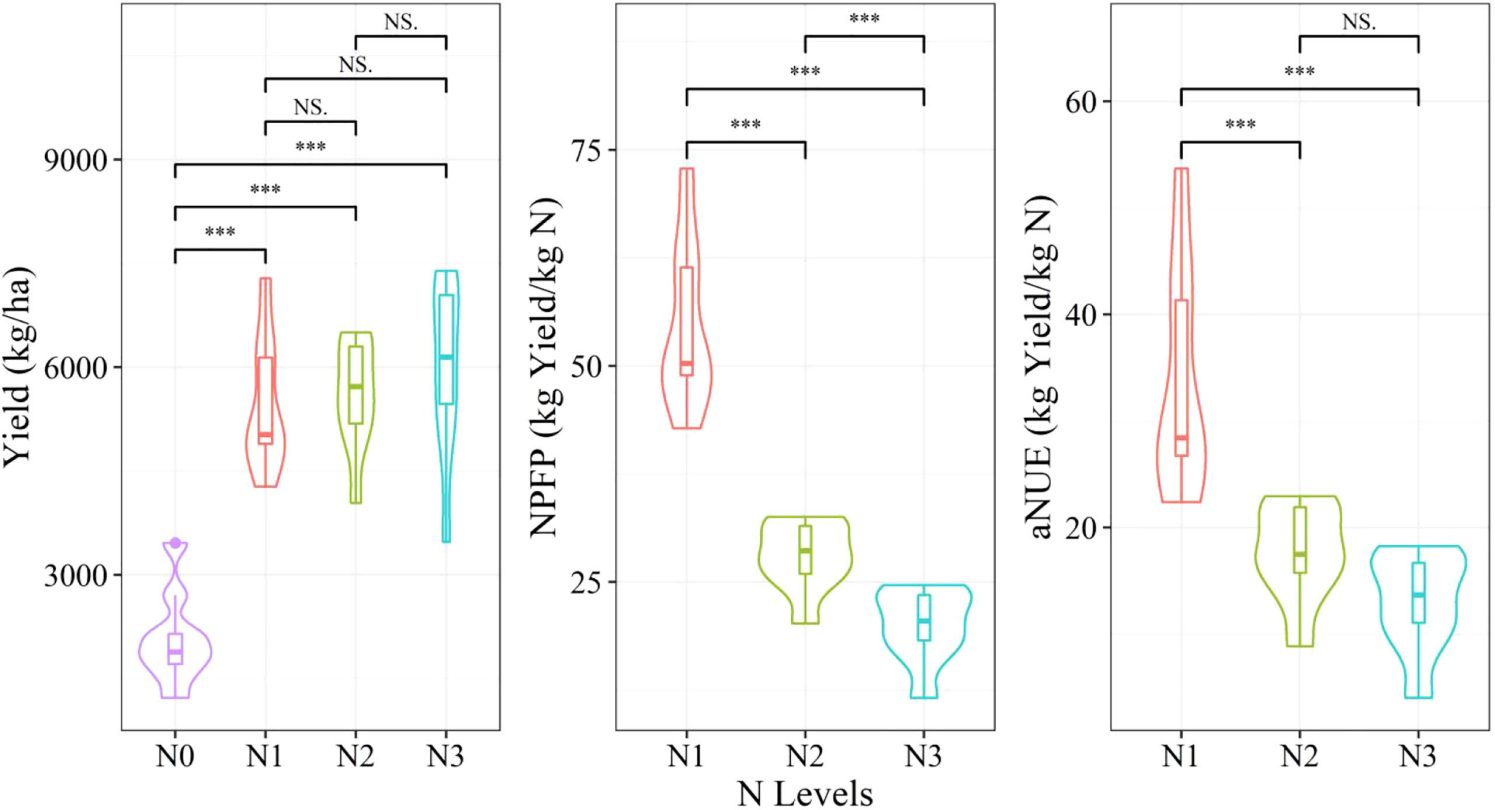
Yield, nitrogen partial factor productivity (NPFP), and agronomical nitrogen use efficiency (aNUE) under different nitrogen levels are shown with the mean and standard deviation. NS, not significant; ***p < 0 by Wilcox’s test.

The winter wheat grain yield and NUE estimation models were established by using the aforementioned four models based on the vegetation indices. Considering the significant differences in the agronomic traits under different nitrogen levels (**Figure 2**), stratified sampling was performed according to the nitrogen levels; two-thirds of the samples were randomly selected as the training set to develop the model, and the remaining one-third of the samples were used as the test dataset for model performance evaluation. The coefficient of determination (R^2^), root mean square error (RMSE), and mean absolute error (MAE) were applied to evaluate the model’s performance. Considering the randomness of sample selection, each model was repeated 20 times to enhance the robustness of the analysis, each time with a different random sampling seed number. The average value of 20 times was considered to evaluate the performance and stability of the model. Specifically, R^2^, RMSE, and MAE were calculated as follows:


(4) $$R^{2}=\frac{\sum_{i=1}^{N}\left(Y_{iact}-\bar{Y_{act}}\right)\left(Y_{ipre}-\bar{Y_{pre}}\right)}{\sqrt{\sum_{i=1}^{N}{\left(Y_{iact}-\bar{Y_{act}}\right)}^{2}{\left(Y_{ipre}-\bar{Y_{pre}}\right)}^{2}}}$$

(5) $$RMSE=\sqrt{\frac{\sum_{i=1}^{N}{\left(Y_{iact}-Y_{ipre}\right)}^{2}}{N}}$$

(6) $$MAE=\frac{1}{N}\sum_{i=1}^{N}\left|\left(Y_{iact}-Y_{ipre}\right)\right|$$

where *Y*_*iact* _ and *Y*_*ipre*_ represent the actual measured values and the predicted values of *i* sample, respectively; $\bar{Y_{act}}$ and $\bar{Y_{pre}}$ represent the average actual measured values and the average predicted values of all samples, respectively; and *N* is the number of samples.

The establishment and evaluation of the above-mentioned estimation models were performed using the RStudio (version 1.4.1106) (https://www.rstudio.com/) with R version 4.04 (https://www.r-project.org/). The four estimation models were implemented using the lm (LR and MLR), lmStepAIC (SMLR), and pls (PLSR) methods in the machine learning package *caret*, respectively. Stratified sampling employed the strata function from the *sampling* package. The PLSR model showed a hyperparameter “ncomp”, which uses a 5-fold cross-validation for determination. To ensure the comparability of the models, the same random number seed was set for each model in each cycle, and the features were standardized.

## Results

### Effect of nitrogen levels on the yield and NUE

**Figure 3:**
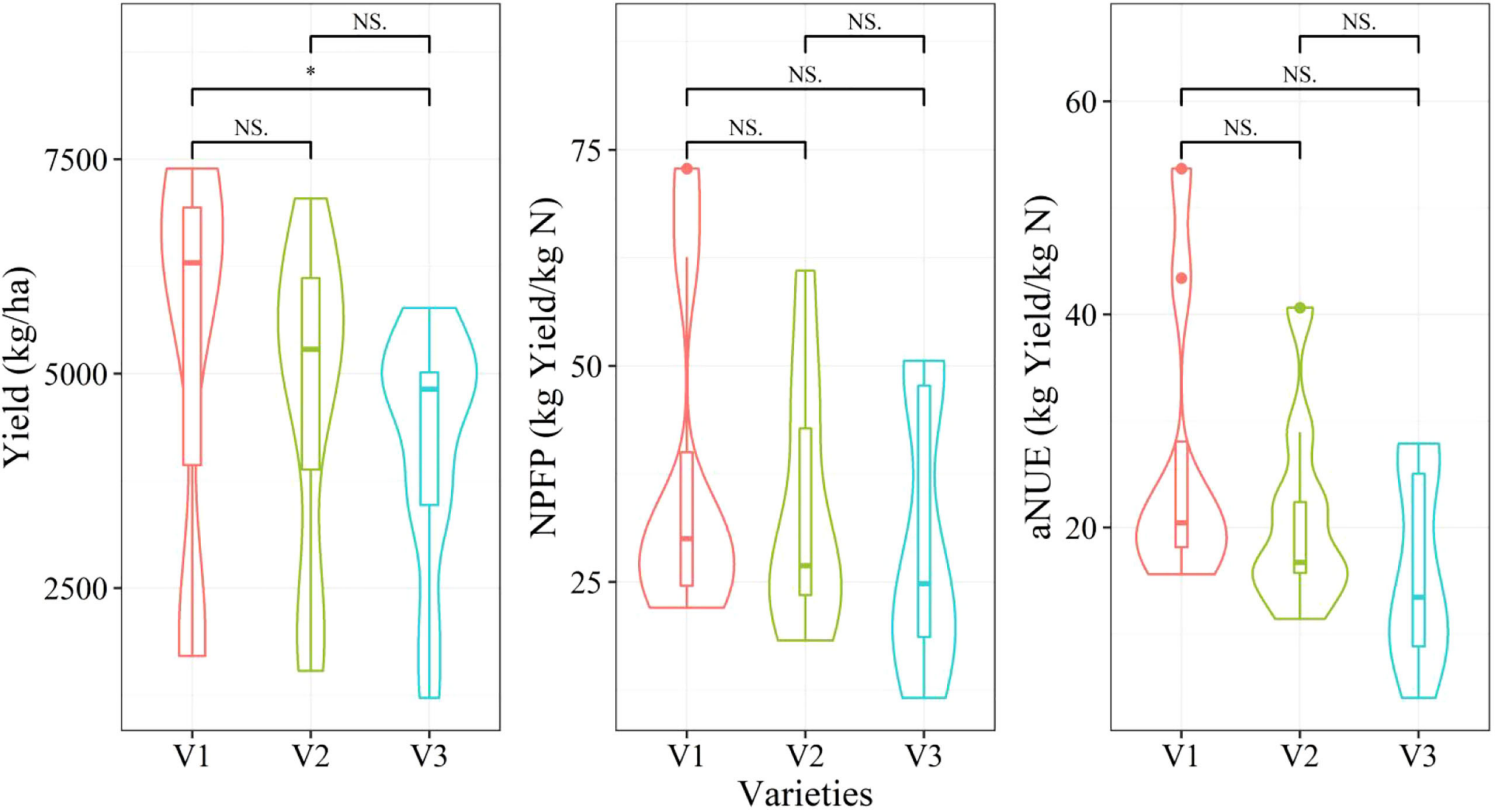
Yield, NPFP, and aNUE under different varieties are shown with the mean and standard deviation. NS, not significant; *p < 0.05 by using Wilcox’s test.

**Figure 2** depicts the effects of different N levels on the yield and NPFP, as well as aNUE. The yield parameter showed an increasing trend with the increase in N levels and varied significantly under N0 and other N treatments. By contrast, both NPFP and aNUE showed a decreasing trend with an increase in N levels, with the former showing a steeper decline. Significant differences were observed in NPFP between different N treatments. For aNUE, significant differences were observed among different N treatments, except for N2 and N3. **Figure 3** depicts the differences in the aforementioned 3 agronomic traits across different varieties. No significant differences were recorded in these parameters among the varieties, except for the yields of varieties V1 and V2, which showed a significant difference. The variety V1 clearly showed the highest yield and NUE, whereas V3 showed the lowest values. In summary, only the nitrogen level was found to significantly affect the yield, NPFP, and aNUE. Therefore, building a predictive model using a stratified sampling strategy is crucial for monitoring wheat yield and NUE under different N treatments.

### Correlations of vegetation indices with yield and NUE

**Figure 4:**
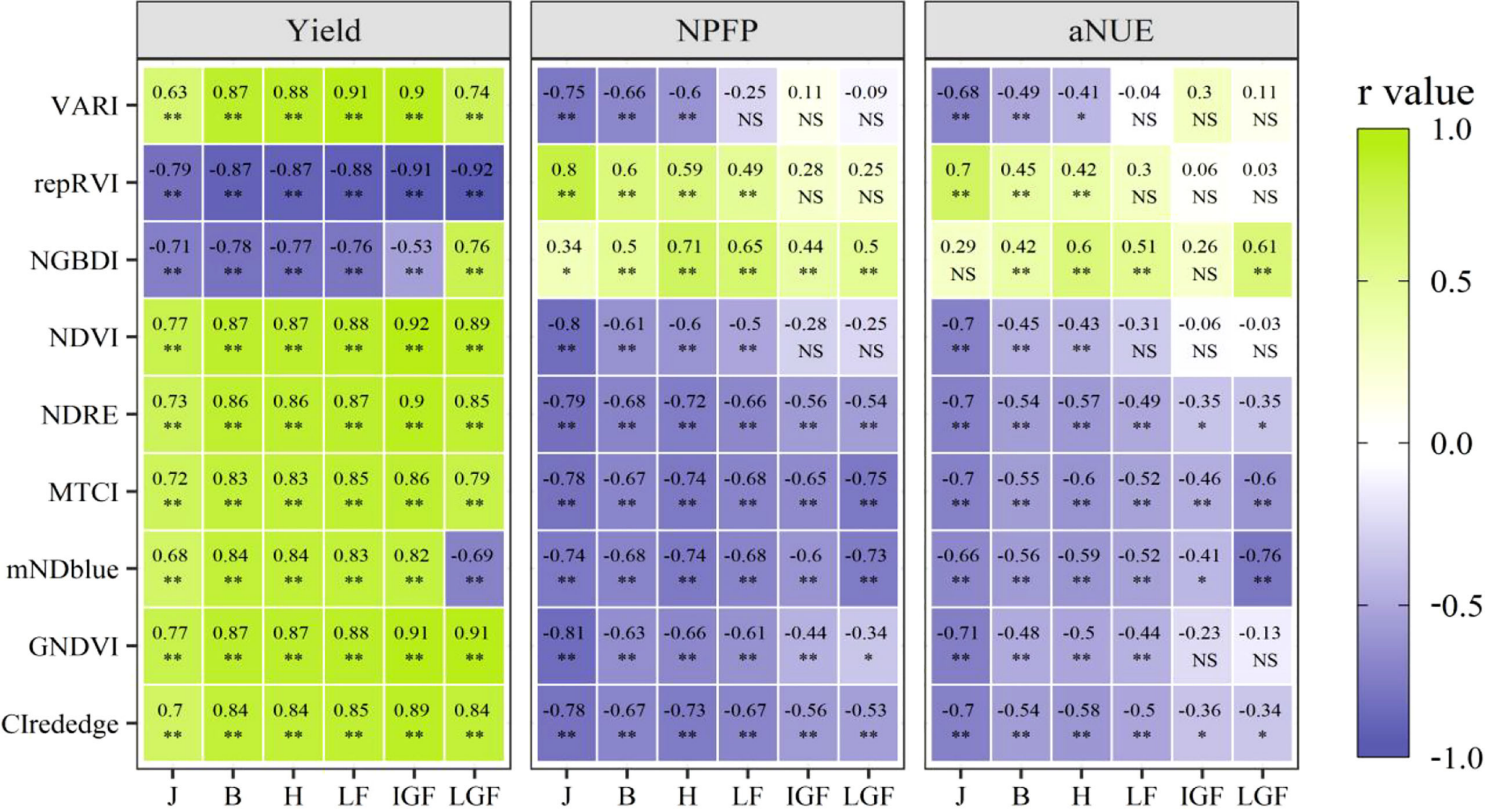
Heatmap for the correlation between vegetation indices and agronomic traits under different growth stages. J, Jointing stage; B, Booting stage; H, Heading stage; LF, Late flowering stage; IGF, Initial grain-filling stage; LGF, Late grain-filling stage; NS, not significant; *: p < 0.05; **: p < 0.01.

Correlation analysis results for the relationship between agronomic traits and multispectral vegetation indices are shown in **Figure 4**. Multispectral vegetation indices significantly are correlated with the yield (r = −0.92 to 0.92) under all N treatments, and the correlation in the middle growth period (from booting to the initial grain-filling stage) was higher than that in the early and late growth periods. repRVI demonstrated strong significant negative correlations in the growing season, with high correlation coefficients ranging from −0.77 to −0.92. It showed the best correlation compared with other indices, particularly in the jointing and late grain-filling stages. NDVI and green normalized difference vegetation index (GNDVI) did not differ from repRVI and were slightly lower than the latter throughout the growing season. NGBDI showed a weak correlation with the yield.

Vegetation indices strongly correlated with NUE at the jointing stage, with NGBDI and GNDVI showing the highest correlation coefficient, followed by repRVI. The correlations of most vegetation indices at the jointing stage were higher than those observed at other stages. However, such a close relationship cannot be synchronized with the growth and development of winter wheat, limiting the indices’ further application in the middle and late growth periods. Throughout the growing season, MTCI and mNDblue showed stable and strong correlations with NPFP (r = −0.65 to −0.78 and r = −0.60 to −0.74, respectively). Notably, the correlation coefficients of these two vegetation indices were much higher than those of the other vegetation indices in the reproductive growth period, and they reached the highest at the maturity stage (r = −0.75 and −0.73, respectively). Middle to strong correlations were also detected between NPFP and other multispectral indices such as NDRE (r = −0.54 to −0.79) and CIrededge (r = −0.53 to −0.78). A similar trend was observed in the correlations between UAVs-based multispectral indices and aNUE. Throughout the growth period, MTCI and mNDblue performed relatively well. Specifically, mNDblue exhibited the highest correlation coefficient (r = −0.76) at the maturity stage. NDRE and CIrededge also achieved middle to strong correlations over the entire season.

### Estimation of yield and NUE for a single critical growth stage

**Figure 5:**
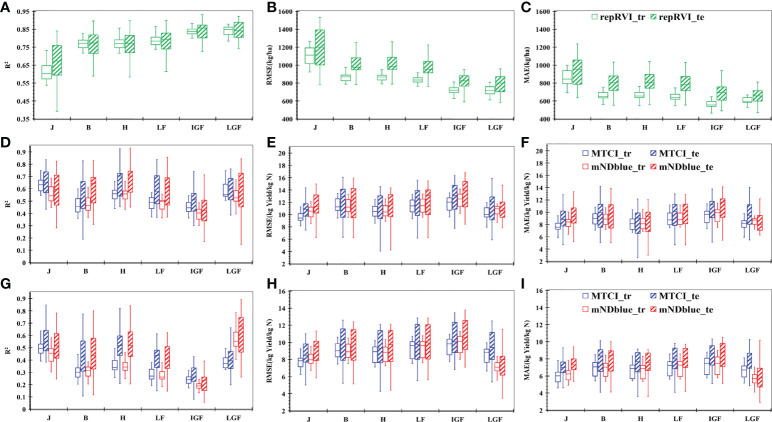
Results of yield **(A-C)** and NUE **(D-I)** prediction for a single critical growth stage using the LR model based on the selected VIs. **(A)** the value of determination coefficient (R^2^) for yield prediction; **(B)** the value of root-mean-squared error (RMSE) for yield prediction; **(C)** the value of mean absolute error(MAE) for yield prediction; **(D)** the value of R^2^ for NPFP prediction; **(E)** the value of RMSE for NPFP prediction; **(F)** the value of MAE for NPFP prediction; **(G)** the value of R^2^ for aNUE prediction; **(H)** the value of RMSE for aNUE prediction, and **(I)** the value of MAE for aNUE prediction. MTCI indicates the MERIS terrestrial chlorophyll index; mNDblue indicates the modified normalized difference blue index; repRVI indicates the reciprocal ratio vegetation index; XXX_tr indicates the result based on the training dataset and XXX vegetation index (RepRVI or MTCI or mNDblue); XXX_te indicates the result based on the test dataset and XXX vegetation index (RepRVI or MTCI or mNDblue). J, Jointing stage; B, Booting stage; H, Heading stage; LF, Late flowering stage; IGF, Initial grain-filling stage; LGF, Late grain-filling stage.

The repRVI presented a high potential for yield assessment throughout the growing season, except for the jointing stage (**Figures 5A–C**). This finding is important for researchers to make better yield prediction before flowering. The best accuracy was achieved at the late grain-filling stage, with R^2^ = 0.85, RMSE = 793.96 kg/ha, and MAE = 654.56 kg/ha.

No significant difference was noted in the predictive performance of MTCI and mNDblue during the entire growth period (**Figures 5D–F**). Considering the stability of the model, the jointing stage was proven to be the optimal stage to conduct NPFP prediction, with R^2^, RMSE, and MAE of 0.65, 10.53 kg yield/kg N, and 8.90 kg yield/kg N, respectively, and followed by the late grain-filling with mNDblue. The initial grain-filling stage showed the worst prediction.

As a similar prediction performance to NPFP, mNDblue provided the best assessment of aNUE (R^2^ = 0.61, RMSE = 7.48 kg yield/kg N, and MAE = 6.05 kg yield/kg N) at the late grain-filling stage (**Figures 5G–I**). MTCI in the jointing stage provided a better prediction, and the worst prediction was obtained in the initial grain-filling stage.

### Estimation of yield and NUE for the vegetative and reproductive growth periods

Spectral information showed apparent differences in vegetative growth and reproductive growth, which are contributed by the difference in the crop structure of the observation field of view. We then analyzed the performance of vegetation indices on yield and NUE prediction in the two periods. No differences were noted in the prediction performance among the three linear models. **Table 4** shows the results of repRVI for winter wheat yield prediction. In the reproductive period, higher performance was achieved, with the average R^2^, RMSE, and MAE of 0.85, 801.05 kg/ha, and 668.53 kg/ha, respectively. Compared with that under the single critical growth stage, the prediction accuracy of yield improved.

**Table 4:** Yield estimation results with multiple linear regression (MLR), stepwise multiple linear regression (SMLR), and partial least-squares regression (PLSR) models for the vegetative and reproductive growth periods based on the repRVI index.

Yield			Vegetative growth period						Reproductive growth period					
			Training dataset			Test dataset			Training dataset			Test dataset		
			R^2^	RMSE	MAE	R^2^	RMSE	MAE	R^2^	RMSE	MAE	R^2^	RMSE	MAE
repRVI	PLSR	mean	0.78	837.84	658.11	0.74	1057.35	859.94	0.87	640.81	512.54	0.85	812.47	676.90
		std	0.04	62.18	51.96	0.09	137.52	134.76	0.03	59.21	44.20	0.06	141.50	128.76
	SMLR	mean	0.78	838.08	657.28	0.74	1045.04	841.67	0.88	622.31	503.42	0.85	803.66	669.09
		std	0.03	60.81	50.95	0.08	125.13	135.77	0.02	52.32	45.07	0.05	151.44	140.85
	MLR	mean	0.79	829.13	655.80	0.74	1060.53	867.07	0.88	618.27	504.36	0.86	787.01	659.61
		std	0.03	59.28	52.05	0.09	130.69	135.65	0.02	51.76	46.03	0.05	146.01	133.61

the unit of RMSE and MAE is kg/ha.

For NUE prediction, MTCI was a better variable in the vegetative period, while mNDblue was anther better variable in the reproductive period (**Tables 5**, **6**). The NUE predicted better on the MTCI and MLR in the vegetative growth period; in the reproductive growth period, the mNDblue and SMLR provided better results for NUE prediction. Similarly, the predictive performance of NUE did not exhibit a significant improvement when compared with that at the single critical growth stage.

**Table 5:** NPFP estimation results with MLR, SMLR, and PLSR models for the vegetative and reproductive growth periods based on the MTCI and mNDblue indices.

NPFP			Vegetative growth period						Reproductive growth period					
			Training dataset			Test dataset			Training dataset			Test dataset		
			R^2^	RMSE	MAE	R^2^	RMSE	MAE	R^2^	RMSE	MAE	R^2^	RMSE	MAE
MTCI	PLSR	mean	0.66	9.11	7.19	0.61	11.34	9.08	0.59	10.10	8.08	0.55	12.05	10.25
		std	0.07	1.15	0.94	0.15	2.26	2.00	0.08	1.39	1.10	0.11	2.99	2.45
	SMLR	mean	0.68	8.94	7.18	0.61	11.25	9.23	0.59	10.03	7.99	0.58	11.46	9.76
		std	0.06	1.12	0.99	0.15	2.59	2.18	0.08	1.33	1.04	0.12	3.14	2.61
	MLR	mean	0.70	8.69	6.97	0.65	10.63	8.65	0.61	9.86	7.79	0.54	11.98	10.05
		std	0.05	0.97	0.85	0.15	2.34	2.08	0.08	1.35	1.00	0.13	3.03	2.48
mNDblue	PLSR	mean	0.64	9.45	7.41	0.59	11.83	9.58	0.69	8.73	6.84	0.61	10.63	8.95
		std	0.08	1.05	0.94	0.15	1.93	1.71	0.07	1.01	0.92	0.18	2.55	2.32
	SMLR	mean	0.68	8.86	7.14	0.62	11.25	9.04	0.69	8.65	6.70	0.64	10.21	8.59
		std	0.04	0.85	0.92	0.16	2.10	1.81	0.07	1.02	0.88	0.19	2.66	2.53
	MLR	mean	0.69	8.81	7.05	0.64	10.97	8.90	0.70	8.56	6.80	0.63	10.38	8.93
		std	0.04	0.83	0.85	0.14	1.95	1.75	0.07	1.03	0.94	0.18	2.59	2.35

the unit of RMSE and MAE is kg yield/kg N.

**Table 6:** aNUE estimation results with MLR, SMLR, and PLSR models for the vegetative and reproductive growth periods based on the MTCI and mNDblue indices.

aNUE			Vegetative growth period,						Reproductive growth period					
			Training dataset			Test dataset			Training dataset			Test dataset		
			R^2^	RMSE	MAE	R^2^	RMSE	MAE	R^2^	RMSE	MAE	R^2^	RMSE	MAE
MTCI	PLSR	mean	0.53	7.39	5.66	0.55	8.84	7.11	0.39	8.50	6.58	0.37	9.97	8.32
		std	0.10	0.94	0.84	0.15	1.94	1.71	0.11	1.25	1.04	0.10	2.29	1.95
	SMLR	mean	0.56	7.22	5.59	0.52	8.84	7.07	0.41	8.35	6.41	0.39	9.87	8.28
		std	0.06	0.89	0.92	0.13	1.76	1.52	0.08	1.09	0.92	0.14	2.38	1.98
	MLR	mean	0.57	7.11	5.48	0.55	8.63	6.81	0.44	8.12	6.15	0.41	9.49	7.67
		std	0.05	0.82	0.86	0.15	1.98	1.78	0.09	1.12	0.91	0.14	2.49	2.09
mNDblue	PLSR	mean	0.49	7.68	5.91	0.50	9.62	7.67	0.59	6.85	5.39	0.52	8.38	7.03
		std	0.11	0.91	0.88	0.18	1.50	1.24	0.09	0.77	0.82	0.22	2.28	2.20
	SMLR	mean	0.56	7.21	5.60	0.49	9.39	7.48	0.59	6.91	5.51	0.55	8.11	6.82
		std	0.06	0.81	0.86	0.17	1.36	1.20	0.10	0.81	0.78	0.24	2.12	2.06
	MLR	mean	0.57	7.12	5.56	0.53	9.04	7.19	0.61	6.69	5.39	0.53	8.30	7.07
		std	0.05	0.73	0.83	0.17	1.54	1.21	0.09	0.79	0.86	0.23	2.31	2.22

the unit of RMSE and MAE is kg yield/kg N.

### Estimation of yield and NUE for the entire growth season

According to the aforementioned results, repRVI, MTCI, and mNDblue showed a good assessment performance for yield and NUE during multiple growth stages, however, the effects of the three linear models in the entire growth season remain unclear.

**Figure 6:**
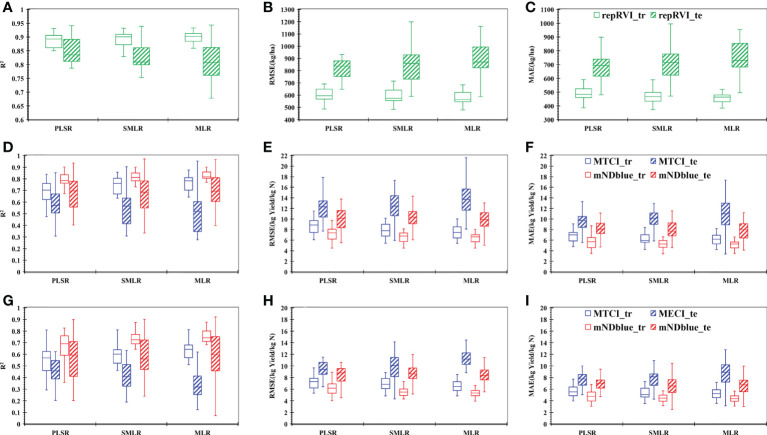
Results of yield **(A-C)** and NUE **(D-I)** prediction with MLR, SMLR, and PLSR models for the entire growing season based on the selected VIs. **(A)** the value of determination coefficient (R^2^) for yield prediction; **(B)** the value of root-mean-squared error (RMSE) for yield prediction; **(C)** the value of mean absolute error(MAE) for yield prediction; **(D)** the value of R^2^ for NPFP prediction; **(E)** the value of RMSE for NPFP prediction; **(F)** the value of MAE for NPFP prediction; **(G)** the value of R^2^ for aNUE prediction; **(H)** the value of RMSE for aNUE prediction, and **(I)** the value of MAE for aNUE prediction. MTCI indicates the MERIS terrestrial chlorophyll index; mNDblue indicates the modified normalized difference blue index; repRVI indicates the reciprocal ratio vegetation index; XXX_tr indicates the result based on the training dataset and XXX vegetation index (RepRVI or MTCI or mNDblue); XXX_te indicates the result based on the test dataset and XXX vegetation index (RepRVI or MTCI or mNDblue). J, Jointing stage; B, Booting stage; H, Heading stage; LF, Late flowering stage; IGF, Initial grain-filling stage; LGF, Late grain-filling stage.

Among the three linear models, the PLSR model achieved the best yield prediction performance (**Figures 6A–C**). The R^2^ of the test dataset was 0.85, the RMSE was 814.61 kg/ha, and the MAE was 642.69 kg/ha, which were comparable to the accuracy of the yield estimation model in the late grain-filling stage.

A similar approach was applied to estimate NUE by using MTCI and mNDblue indices. mNDblue performed significantly better than MTCI (**Figures 6D–I**). Although the three linear models did not show differences, better results for NPFP assessment were achieved based on the mNDblue and MLR, with R^2^, RMSE, and MAE of 0.70, 9.59 kg yield/kg N, and 7.70 kg yield/kg N, respectively (**Figures 6D–F**). This result outperformed the prediction results obtained using only the critical growth stage, as shown in **Figure 5**.

Similar to the results of NPFP, no noticeable difference was noted among the three linear models, and the best results were also obtained based on the mNDblue and MLR (R^2^ = 0.60, RMSE = 8.11 kg yield/kg N, and MAE = 6.58 kg yield/kg N) for aNUE (**Figures 6G–I**), which were comparable to the results at the late grain-filling stage.

### Estimation of yield and NUE using all vegetation indices

**Figure 7:**
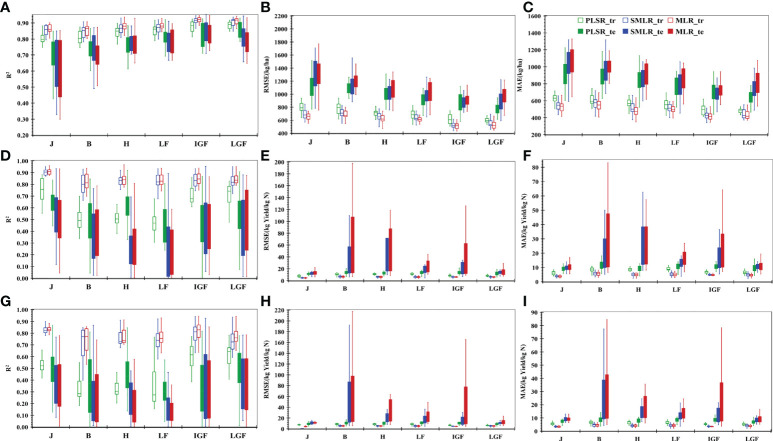
Results of yield **(A-C)** and NUE **(D-I)** prediction with MLR, SMLR, and PLSR models at different stages based on all vegetation indices. **(A)** the value of determination coefficient (R^2^) for yield prediction; **(B)** the value of root-mean-squared error (RMSE) for yield prediction; **(C)** the value of mean absolute error(MAE) for yield prediction; **(D)** the value of R^2^ for NPFP prediction; **(E)** the value of RMSE for NPFP prediction; **(F)** the value of MAE for NPFP prediction; **(G)** the value of R^2^ for aNUE prediction; **(H)** the value of RMSE for aNUE prediction, and **(I)** the value of MAE for aNUE prediction. MTCI indicates the MERIS terrestrial chlorophyll index; mNDblue indicates the modified normalized difference blue index; repRVI indicates the reciprocal ratio vegetation index; XXX_tr indicates the result based on the training dataset and XXX vegetation index (RepRVI or MTCI or mNDblue); XXX_te indicates the result based on the test dataset and XXX vegetation index (RepRVI or MTCI or mNDblue). J, Jointing stage; B, Booting stage; H, Heading stage; LF, Late flowering stage; IGF, Initial grain-filling stage; LGF, Late grain-filling stage.

Further analysis was performed to determine whether redundant independent variables affect the prediction results. **Figures 7A–C** describes the effect of all VIs on the yield, indicating that the yield assessment performance of SMLR and MLR increased from the jointing stage to the initial grain-filling stage and then decreased at the late grain-filling stage. Taking MLR as an example, the R^2^ (RMSE and MAE) values changed from 0.62 (1322.23 kg/ha and 1055.07 kg/ha) to 0.82 (900.71 kg/ha and 725.50 kg/ha), which then fluctuated to 0.78 (989.24 kg/ha and 811.37 kg/ha). Conversely, the prediction result of PLSR was satisfactory with R^2^, RMSE, and MAE of 0.70, 1120.59 kg/ha, and 908.77 kg/ha, respectively, at the jointing stage, which then gradually increased to 0.86, 776.83 kg/ha, and 642.69 kg/ha, respectively. The performance was slightly improved when compared to a linear model based on a single critical stage by using repRVI.

**Figures 7D–I** depicts the prediction performance for NUE of all vegetation indices at different stages in terms of R^2^. Although the PLSR model showed a lower accuracy than SMLR and MLR models on the training dataset than SMLR and MLR, the accuracy on the test dataset was better than with the other two models, and the R^2^ distribution was concentrated. In short, the PLSR model was more effective in overcoming the problem of unbalanced prediction accuracy on the test dataset and the training datasets caused by overfitting and performed better on the test dataset. Compared with the single-stage estimation model, the prediction performance based on all VIs did not improve. These results suggested that improving the prediction performance by increasing the number of input features is not necessarily a good choice.

## Discussions

### Reliability of the P4M camera

UAVs are increasingly being used for crop growth monitoring and field phenotyping. In the recent decade, UAVs have entered the consumer goods market in parallel with the continuous development of low-cost sensing technology. The appearance of DJI P4M has made the multispectral remote-sensing system available in the market. Although there are cheap CIR cameras that can capture NIR images by modifying the images obtained through ordinary RGB cameras, the red-edge band, which is of great significance to crop monitoring, remains a luxury. Nevertheless, P4M integrates this band to the consumer level, which positively promotes the significance of the popularization of agricultural remote sensing. It is especially well-received by users with limited funds. Lu et al. (2020) compared the consistency of the spectral features of Parrot Sequoia and P4M and noted a high correlation among the green, red, red-edge, and near-infrared bands. In their study, the consistency between P4M-NDVI and the ground measured ASD_NDVI was compared to reveal a correlation coefficient of >0.85. However, it remains questionable that the processing of P4M reflectance in the study was replaced by dividing 10^5^ based on the original DN value, which lacked radiometric calibration in remote sensing. It is well-known that NDVI is calculated based on reflectance rather than DN value. Although satisfactory results were obtained in the study Lu et al. (2020), the methods used for data processing remain questionable. A recent study (Di Gennaro et al., 2022) discussed the exposure mode and radiometric calibration of P4M in detail and exhibited that irrespective of whether the exposure mode was set manually or automatically, there was no difference in data obtained and users can obtain professional-quality data without any background in optics. On the other hand, they compared the influence of 4 radiometric calibration methods (M1-M4 for short; M1 and M2 performed no radiometric calibration, while M3 and M4 performed radiometric calibration with ELM) on the accuracy of vegetation indices. Their results showed that M3 (empirical linear correction performed on the orthophoto images by the DJI Terra software with multiple reference reflectance panels of known reflectance) had high accuracy. The authors also reported that the accuracy in the crop canopy of the VIs calculated after the correction by this method was equivalent to that of the improved Micasense RedEdge camera (Lu et al., 2020). These results mainly suggest the spectral reliability of the P4M camera and its effectiveness in vegetation monitoring. However, another ambiguity is raised as a result of the use of VIs instead of band reflectance values by the authors during the authenticity test of the spectral performance of P4M. It is well-known that band reflectance is the basis for calculating spectral VIs. If the authenticity of band reflectance can be verified, numerous VIs can be calculated or created according to the corresponding formula to meet the diversified agricultural application scenarios. Another consideration was that although three VIs were used in this study, more than 50 VIs calculated based on reflectance were initially referenced (data not shown). The comparison of the spectral consistency among a few VIs in past studies could not cover this huge index group. Therefore, the reflectance of the P4M bands was compared with the measured reflectance of the ground ASD HH2 spectrometer to establish a solid theoretical basis for further research, as well as for its wider and deeper application in the future.

**Figure 8:**
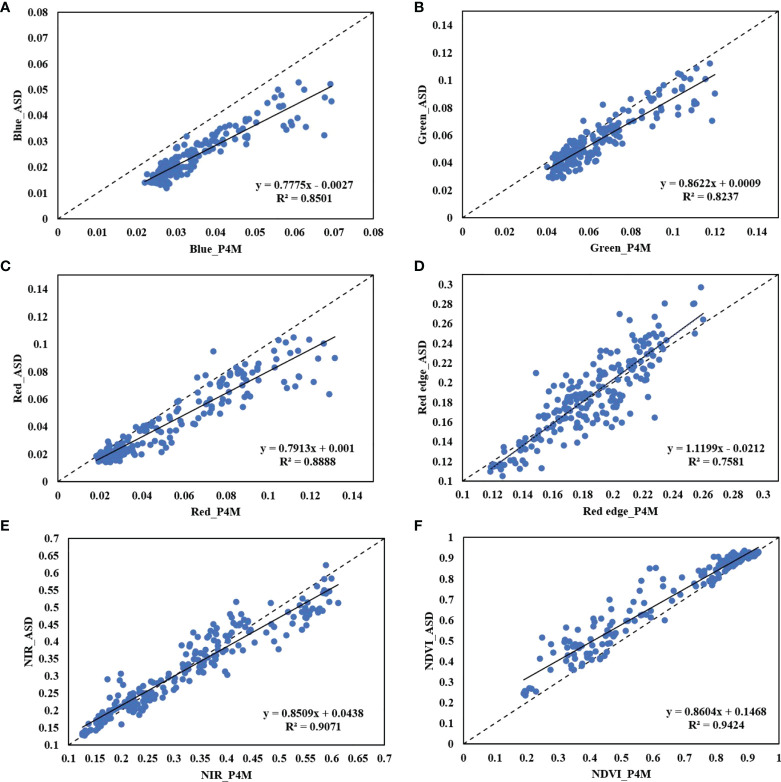
Analysis of the correlation between the reflectance of P4M bands and ground measured reflectance of the ASD HH2 spectrometer **(A–E)** and corresponding NDVI **(F)**. The dotted line indicates a 1:1 line.

**Figures 8A–E** shows the scatter distribution of the reflectance of P4M bands after radiometric calibration and the mean reflectance measured by the ground ASD HH2 spectrometer, respectively. The correlation between the reflectance of the remaining four P4M bands, except for the red-edge band, and the measured reflectance exceeded R^2^ = 0.82. The correlation between the red and near-infrared bands was higher, and the scatter distribution was closer to the 1:1 line. The comparison of NDVI also suggested a significant correlation with R^2^ = 0.94 (**Figure 8F**), which is higher than that reported by Lu et al. (2020) (R^2^ = 0.88). This may benefit from the efficient radiometric calibration method adopted in this study. Compared with the results of Di Gennaro et al. (2022), the NDVI in this study also obtained a good percentage error (PE) in the wheat canopy (this study: PE = 9.5%, the study of Di et al.: PE = 9.9%). The correlation between the reflectance of the red-edge band and the ground measured reflectance was the lowest. The NDRE calculated by the calibrated red-edge reflectance and near-infrared reflectance elsewhere (Di Gennaro et al., 2022) showed poor PE accuracy (PE = 19.4%), however, the authors did not analyse this result further. We believe that this can be deemed to be associated with the setting of the red-edge band with a bandwidth of 32 nm, which is greater than the setting of similar multispectral cameras (**Supplementary Table S2**). A large bandwidth setting contributes to imprecise determination of the position of the red edge. The red edge may get missed at the steepest slope of the sharp rise region of the typical green vegetation reflectance spectrum from the red light to near-infrared light. Furthermore, Holman et al. (2019) demonstrated that the spectral reflectance of 30 wheat varieties obtained by the Parrot Sequoia camera under 4 nitrogen treatments was compared to that measured by the Tec5 HandySpec Field Spectrometer. Holman et al. (2019) reported that the correlation of the NIR band with R^2^ = 0.74, which is consistent with that of the red-edge band with R^2^ = 0.76 in this study. In comparison with the wide application of the Parrot Sequoia camera in agricultural research, the correlation of the red-edge band of P4M was within the acceptable range. Moreover, P4M has been used in several studies with ambiguous calibration methods to obtain data, which further proves the effectiveness of this camera (Gallardo-Salazar and Pompa-García, 2020). Considering that this study is the first to verify the accuracy of the reflectance of the P4M bands from the perspective of reflectance, whether the weak correlation of the red-edge band is an exception remains unestablished. Further work is needed in this direction to validate the current findings.

### Physiological interpretation of UAVs-based VIs

VIs shows good potential in crop growth monitoring by UAVs (Yang and Guo, 2008; Qiu et al., 2018). Using suitable VIs, instead of the complex methods based on VIs, is the key for agronomic trait estimation. This study developed generic evaluation models for early yield and NUE monitoring based on three promising VIs that can be applied to multiple growth stages during the growing season of wheat. One of these VIs — repRVI, was an index of yield; the other two indices, MTCI, and mNDblue, showed relatively consistent performance in the estimation of two NUE traits.

The results revealed that repRVI performed best in grain yield assessment at the LGF stage, which is consistent with previous studies (Hassan et al., 2019; Fei et al., 2021a; Fei et al., 2021b; Ganeva et al., 2022), because the LGF stage is close to maturity and the information in the UAVs field of view is mainly provided by the mature spikes. The signal is minimally affected by moisture and other green parts of the rice plant.

Furthermore, repRVI performed best at the LGF stage, which may be related to the construction of this index. The green signal is relatively less prominent and the reflectance of the red band is relatively higher in LGF stage. The high value of Red band makes the NIR/Red value lower overall when Red band is used as the denominator, but the fact that the magnitude of NIR band is more than 10 times higher than that of the Red band cannot be ignored, thus, the NIR/Red is more dependent on the numerator NIR, which makes it difficult to effectively capture the weak changes brought about by the elevation of Red band. However, the repRVI uses Red band as a molecule and NIR band as a denominator, which can better capture rising trends of Red band and is sensitive to weak information. Thus, the repRVI index is more conducive to monitoring vegetation information in the grain-filling stage.

The MTCI and mNDblue performed stably and demonstrated significant correlations with NUE throughout the growing season. Another interesting finding is that MTCI was more suitable for NUE prediction in the vegetative growth period, while mNDblue was more suitable for evaluation in the reproductive growth period. Chlorophyll is closely related to nitrogen, and high chlorophyll and N contents in the plant are obvious signs of effective nitrogen fertilization supply (Tian et al., 2011). Due to the red edge band, MTCI is sensitive to the chlorophyll content and should not be saturated at a high chlorophyll concentration (Dash and Curran, 2004). At a high chlorophyll concentration in the vegetative growth stage, other vegetative indices may be saturated to varying degrees, whereas MTCI can help avoid this problem and thus better reflect the photosynthetic capacity of rice, which is conducive to adequate nitrogen uptake and increased yield (Tian et al., 2011). mNDblue was improved by Jay et al. (2017), who found that mNDblue was sensitive to canopy chlorophyll content when the effect of soil background is weak. During the reproductive growth period of winter wheat, the effect of soil background was minimized, thus maximizing the performance of the mNDblue. We also found correlation coefficient of more than 0.97 between the two indices, which may explain the similarity and difference in NUE prediction by these two indices.

Furthermore, we also calculated the weighted mean/maximum-rank sums (WMMRS) score, which evaluates the optimum vegetation index that is robust towards date-specifific effects through linear relationship (Prey et al., 2020) for 9 vegetation indices. These three indices obtained the highest scores among all indices in terms of generalized performance throughout the whole growing season (**Supplementary Table S3** and **Appendix document A**), which is a major motivation for our selection.

### Accuracy and stability of yield and NUE prediction models

In recent years, several studies on the yield and/or NUE of different genotypes of crops based on remote sensing have proved the applicability of UAVs in the field. The technical system for yield prediction is mature and advanced, however, the current studies on NUE have been limited in quantity or quality. Only one study used the UAVs to evaluate the NUE of winter wheat (Yang et al. 2020). Notably, NUE was distinguished from the nitrogen content. Although the nitrogen content in plant reflects the ability of a crop to absorb nitrogen, NUE emphasizes the extent to which crops can utilize external nitrogen applications.

Our study showed that the model has high accuracy for yield prediction and moderate accuracy for NUE prediction. Despite a gap between NUE prediction accuracy and yield prediction accuracy, the models have equivalent or higher accuracy compared with those reported in similar studies (**Table 7**).

**Table 7:** Previous research results similar to those of the present study.

Platforms	Traits	Sensors	Crops	Stage	Best results	Reference
Ground	Yield\NutEff_grain	PhenoTrac 4 multi-sensor	wheat	initial grain-filling	LR: Yield:R^2^ = 0.52; NutEff_grain: R^2^ = 0.21 PLSR: Yield:R^2^ = 0.33 RMSE = 1891kg/ha	Prey et al., 2019
	Yield\NUtE	Ocean Optics USB2000 spectrometers	wheat	heading to initial grain-filling	LR: Yield: R^2^ = 0.47; NUtE: R^2^ = 0.44 PLSR: Yield: R^2^ = 0.42,RMSE = 792kg/ha; NUtE: R^2^ = 0.41,RMSE = 2.2	Pavuluri et al., 2015
	Yield\NPFP	JAZ portable field spectrometer	wheat	heading	MLR: Yield: R^2^ = 0.83,RMSE = 565.29kg/ha MLR: NPFP: R^2^ = 0.85 under low N conditions MLR: NPFP: R^2^ = 0.77 under normal N conditions	Frels et al., 2018
UAVs	Yield \aNUE\NPFP	Tetracam mini MCA	barley	grain-filling	MLR: Yield: R^2^ = 0.827	Kefauver et al., 2017
	aNUE	Parrot sequoia	wheat	mid to late grain-filling	LR: R^2^ = 0.78, RMSE = 0.004	Yang et al. 2020
	Yield	Micasens- RedEdge-M	wheat	initial grain-filling	SVR: R^2^ = 0.91, MSE = 0.14	Shafiee et al., 2021
	Yield	Airphen	wheat	whole	RF: R^2^ = 0.78, RMSE =103 kg/ha	Fu et al., 2020
	Yield	Parrot Sequoia	wheat	tillering and grain-filling	LR: R^2^ = 0.62, RMSE = 972 kg/ha	Zhou et al., 2021
	Yield	Parrot Sequoia	wheat	late grain-filling	SLR: R^2^ = 0.89	Hassan et al., 2019
	Yield	MultiSPEC 4C	wheat	heading to grain-filling	MLR: R^2^ = 0.70, RMSE = 618.30 kg/ha	Zhu et al., 2018
	Yield	multispectral camera MQ022MG-CM	rice	whole	RF: R^2^ = 0.78, RMSEP = 370 kg/ha, rRMSE = 3.20%	Wan et al., 2020
	Yield	Tetracam mini MCA	rice	booting and heading	MLR: R^2^ = 0.75, RMSE = 926.46 kg/ha	Zhou et al., 2017
	Yield	Parrot Sequoia	maize	anthesis	ANN: R^2^ = 0.97, RMSE = 425kg/ha, MAE = 249kg/ha	García-Martínez et al., 2020

NutEff_grain indicates the grain N utilization efficiency; NutE indicates N utilization efficiency; LR indicates linear regression; MLR indicates multiple linear regression; SMLR indicates stepwise multiple linear regression; PLSR indicates partial least-squares regression; RF indicates the random forest; SVR indicates support vector machine regression, and ANN indicates artificial neural network regression.

The results of yield prediction were compared with those of past studies, and the present study results are equivalent to or better than the prediction results of wheat yield reported in previous studies (**Table 7**). We believe that the main difference between these studies with higher accuracy was that the input features not limited to VIs, plant height, canopy coverage, or density were included and the machine learning method was applied in these studies (Fu et al., 2020; García-Martínez et al., 2020; Klompenburg et al., 2020; Wan et al., 2020; Shafiee et al., 2021). For instance, previous studies have used the RF model (Fu et al., 2020; Wan et al., 2020), the neural network model (García-Martínez et al., 2020), and SVR regression models (Shafiee et al., 2021).

Regarding the prediction of NUE, the values reported in this study are lower than those reported by Yang et al. (2020) and Pavuluri et al. (2015) (**Table 7**). Unlike the approach adopted in these studies, different N treatments were simultaneously considered in our study to establish models. Another explanation for this was the significant difference in the wheat genotype used in these studies. However, there was no difference in the NUE parameters of the three varieties in the present study. The NDRE at the middle and late grain-filling stages in a past study (Yang et al., 2020) showed the best mean R^2^ and RMSE. NDRE was also applied in this study, which showed a moderate correlation with NUE. The MTCI was also considered in the studies by Prey et al. (2020) and Frels et al. (2018); however, it showed the worst performance on the test dataset. This result can be explained by three possible reasons: (1) the dependent variable in the study Fresl et al. (2018) was NutE (NutEff_grain). Although it also emphasized NUE, the calculation method was different (yield/nitrogen uptake), with the denominator being the plant nitrogen content, rather than the amount of fertilizer applied; (2) the MTCI was first developed based on broadband and was calculated by the reflectance in the narrow hyperspectral bands in these studies (Frels et al., 2018; Prey et al., 2020); (3) several wheat genotypes (22–75 genotypes) have been used in past studies (Pavuluri et al., 2015; Prey et al., 2020), which could have resulted in low R^2^.

Machine learning is being increasingly applied in the estimation of crop parameters (Jin et al., 2021). It complements big data and high-performance computing. We used only 3 varieties and 4 nitrogen levels in this study, totaling 36 samples with only 9 features involved, which did not show the characteristics of high-dimensional data. Hence, significant improvements may not be necessarily obtained by machine learning, as also verified by Zhou et al. (2021). Another key consideration is that machine learning is in a black box. Consequently, there are certain obstacles for personnel without a background in data analysis science, which limits its further popularization. If the applied research remains limited to the laboratory level, its value will be greatly reduced (Klompenburg et al., 2020). Considering the aim of this study was to further verify the reliability of P4M camera for yield and NUE rapidly evaluation and to provide a cost-effective and practical approach for agricultural practitioners lacking remote sensing experience, several understandable and operatable linear models were used in this study. The results support our choices.

According to the results depicted in **Figures 2**, **3**, the three agronomic parameters were sensitive to the nitrogen level but not to the variety. Therefore, when establishing regression models, varieties were not differentiated. Stratified sampling was adopted when dividing the training and test datasets according to the nitrogen level. It allowed the division of an equal proportion of samples for each nitrogen level to participate in modeling. Regression models were established according to the nitrogen level as in a past study to obtain more accurate models. However, the sample size was smaller (N = 9). The models at the middle and late grain-filling stages adopted elsewhere (Yang et al. 2020) used 9 samples (3 samples/year), and the training dataset was not distinguished from the test dataset. The stability and applicability of such models are uncertain. To establish a more robust model, we adopted 20 cycles in this study to calculate the average value of the model evaluation indices to display the stability of the model. The modeling results showed that the training dataset was much more stable than the test dataset. When more independent variables were input, the PLSR model was more stable and had fewer outliers because the PLSR model could better deal with the collinearity of multidimensional variables. Generally, the stability of the prediction model for yield was higher than that for NUE. The robustness of the prediction model trained by multiple cycles has rarely been examined in past studies. Our study demonstrated that the error of model performance caused by the difference in random sampling was also worth considering. Random sampling is approximately unbiased only when the population is sufficiently large. For a small sample size, it cannot be ensured whether the samples collected each time are unbiased or depict an approximately unbiased estimate of the population. Therefore, confirming the stability of the model by increase the number of sampling cycles for a small sample size is highly recommended.

### Implications for future work

Compared with ground assessment, the non-destructive UAVs remote-sensing evaluation is repeatable and flexible and provides real-time data. The popularity of the consumer drone market has further promoted the in-depth application of UAVs remote-sensing technology in the field of agricultural research, however, only a few studies have used P4M cameras to predict wheat yield and nitrogen use efficiency. Our research aims to explore and establish a general strategy for better prediction of yield and NUE across multiple growth periods, thereby providing a low-cost data analysis strategy for potential non-expert users of consumer-grade multispectral UAVs. In this study, three P4M-based vegetation indices with good performance were proposed. The combination of these vegetation indices and linear models can provide a rapid and cost-effective method to assess yield and NUE, demonstrating the great potential of P4M camera for quantifying important crop traits.

Although our model based on three genotypes adequately accounted for the differences in varieties and nitrogen levels, the performance of the model must be tested on multiple wheat varieties where the differences between varieties were more significant. The model can be applied in practice and accepted by researchers and applicators only by developing it as a general prediction model for yield and NUE assessments by considering different N gradients and varieties. In addition, continued multi-year trials are warranted, and future attention should be paid to seasonal differences in the proposed general strategy to compensate for the lack of inter-year variation characteristics in the current study. Considering the increasing application of UAVs remote sensing in agriculture, developing a comprehensive and shared database to support mutual verification of the same research purposes and future in-depth exploration of precision agriculture is essential.

## Conclusions

In this study, we explored the potential of a consumer-grade multispectral P4M camera for monitoring the winter wheat grain yield and NUE traits. For this purpose, three universal vegetation indices showing high correlations with the target-dependent variables were determined. The results revealed that the repRVI presented a high potential for grain yield assessment during the entire growing season, except at the jointing stage. The late grain-filling stage was identified as the optimal single stage to predict the grain yield and achieve the prediction results, with R^2^ = 0.85, RMSE = 793.96 kg/ha, and MAE = 656.31 kg/ha. The performance of the yield estimation combining multiple stages improved slightly but not significantly. Both MTCI and mNDblue exhibited a significant correlation with NUE. The simple LR model based on the MTCI index at the jointing stage showed a good performance in NPFP assessment, with R^2^ = 0.65, RMSE = 10.53 kg yield/kg N, and MAE = 8.90 kg yield/kg N, followed by the mNDblue at the late grain-filling stage. Good performance with the mNDblue index for aNUE was observed at the late grain-filling stage, with R^2^ = 0.61, RMSE = 7.48 kg yield/kg N, and MAE = 6.05 kg yield/kg N, followed by MTCI at the jointing stage. Combining multiple stages did not improve NUE traits assessment accuracy. Moreover, MTCI and mNDblue were suitable for NUE prediction in the vegetative growth period and reproductive growth period, respectively.

The differences among the three linear models became evident with an increase in the number of input independent variables. The PLSR model with all VIs as input features showed better robustness than the other regression models, albeit the accuracy did not improve noticeably.

Importantly, our study demonstrated the effectiveness of the DJI P4M camera as a high-throughput phenotyping platform for small-scale crop monitoring tasks, and the selected indices can serve as effective indicators for timely and accurate prediction of yield and NUE prior to harvest. In the future research, the potential of P4M camera in different climates, seasons, and varieties should be studied and the capabilities of UAV remote sensing for diversified agricultural applications should be thoroughly explored.

## Data availability statement

The original contributions presented in the study are included in the article/**Supplementary Materials**. Further inquiries can be directed to the corresponding author. The Supplementary material and Appendix document for this article can be found online at: https://figshare.com/s/fa258c55dd6bd9fc69b9 named as Supplementary Material.zip.

## Author contributions

XL, JL, and YZ designed and developed the research idea. YZ, XC, and XT conducted the field data collection. JL and YZ performed the data analysis. JL wrote the manuscript. JL, YZ, XT, XC, and XL contributed to the results and data interpretation, discussion, and revision of the manuscript. All the authors revised and approved the manuscript. All authors have read and agreed to the published version of the manuscript.

## Funding

This research were funded by the Xinjiang Construction Corps Oasis Ecology Key Laboratory Open Project Development Fund Project (201903), a university-level scientific research project of Anhui Science and Technology University (2021gnny02). Major science and technology projects of Anhui Province of China (201903a06020001) and Sub-project under National Science and Technology Support Program of China (2018YFD0300901-2).

## Acknowledgments

We are very grateful to Jun Li, Xueqing Zhu, and Qingyang Liu for their assistance in data collection and we would also like to thank Jie Liu and Fang Gao for their valuable suggestions on the paper.

## Conflict of interest

The authors declare that the research was conducted in the absence of any commercial or financial relationships that could be construed as a potential conflict of interest.

## Publisher’s note

All claims expressed in this article are solely those of the authors and do not necessarily represent those of their affiliated organizations, or those of the publisher, the editors and the reviewers. Any product that may be evaluated in this article, or claim that may be made by its manufacturer, is not guaranteed or endorsed by the publisher.
