# Supplementary material for: Rapid prediction of winter wheat yield and nitrogen use efficiency using consumer-grade unmanned aerial vehicles multispectral imagery
Source: Front Plant Sci. 2022 Oct 24;13:1032170. doi: 10.3389/fpls.2022.1032170 (PMC9638066; doi:10.3389/fpls.2022.1032170)
Supplement: Supplementary file 1 [file DataSheet_1.docx]

Supplementary Material

**TABLE S1** Winter wheat accuracy evaluation after background removal based on VARI threshold

| Stages | User's accuracy(%) | Producer's accuracy(%) | Over accuracy(%) | Kappa | Threshold |
| --- | --- | --- | --- | --- | --- |
| J | 99.79 | 99.69 | 99.65 | 0.99 | 0.07 |
| B | 98.79 | 98.30 | 98.06 | 0.96 | 0.13 |
| H | 99.24 | 98.14 | 98.25 | 0.96 | 0.08 |
| LF | 99.22 | 98.56 | 98.51 | 0.97 | 0.05 |
| IGF | 98.72 | 97.97 | 97.79 | 0.95 | 0.01 |
| LGF | 99.53 | 99.60 | 99.42 | 0.99 | * |

Note: Using the classification results of the Jointing stage.

Referring to past research (Jay et al., 2019), we selected the visible atmospherically resistant index (VARI) and used the threshold method to remove the soil background. The pixels of wheat and soil background were delineated through UAV remote sensing images and field surveys, after which the VARI value was calculated, and the threshold was set through repeated experiments. Thresholds dynamically changed in various stages. Winter wheat accuracy evaluation based on VARI threshold was detailed in Table S1. It should be noted that the accuracy of the VARI threshold method was less at late grain filling (LGF) stage. We also tried support vector machine classification; however, the results were not satisfactory. Finally, we directly applied the segmentation results from other first 5 stages to LGF and selected the segmentation results of the jointing period with the highest accuracy as the final result of LGF stage. Comparing the results from the OSTU threshold method, the results from the current study achieved better accuracy in both visual effects and confusion matrix validation in all stages.

**TABLE S2** Comparison of four multispectral cameras applied in agricultural remote sensing

| Sensors  Bands | RedEdge^1^ | P4M^2^ | Airphen^3^ | Parrot Sequoia^4^ | MQ022MG-CM^5^ | SparkFun number SEN-14351^6^ |
| --- | --- | --- | --- | --- | --- | --- |
| blue | 475nm±16nm | 450nm±16nm | 450nm±8nm |  | 25 wavebands in the spectral region of 600–1000 nm |  |
| green | 560nm±13.5nm | 560nm±16nm | 560nm±8nm | 550nm±20nm |  |  |
| red | 668nm±8nm | 650nm±16nm | 650nm±8nm | 660nm±20nm |  | 680nm±20nm |
| red edge | 717nm±6nm | 730nm±16nm | 730nm±8nm | 735nm±5nm |  | 730nm±20nm |
| NIR | 842nm±28.5nm | 840nm±26nm | 840nm±13nm | 790nm±20nm |  | 810nm±20nm |
|  |  |  |  |  |  | 860nm±20nm |
| Resolution (px*px) | 1280 × 960 | 1600×1300 | 1280 × 960 | 1280 × 960 | 2048 × 1088 |  |
| Reference | Fei et al., 2021a, 2021b;  Shafiee et al., 2021 | Bruce et al., 2021;  Di Gennaro et al., 2022; Zhang et al., 2022; | Jin et al., 2017;  Jay et al., 2019;  Fu et al., 2020; | Han et al., 2019;  Hassan et al., 2019;  Yang et al., 2020;  Alabi et al., 2022 | Wan et al., 2020 | Habibulla et al., 2020 |

Note: 1. https://micasense.com/zh-hant/rededge-mx/;

2. https://www.dji.com/cn/p4-multispectral/specs;

3. https://www.hiphen-plant.com/;

4. <https://www.parrot.com/us/support/documentation/sequoia>.

5. <https://www.ximea.com/en/products/usb3-vision-cameras-xiq-line/mq022mg-cm>

6. <https://www.digikey.com/en/products/detail/sparkfun-electronics/SEN-14351/7803409>

**TABLE S3** Weighted mean/maximum rank sums (WMMRS) for all VIs.

| WMMRS | VARI | repRVI | NGBDI | NDVI | NDRE | MTCI | mNDblue | GNDVI | CIrededge |
| --- | --- | --- | --- | --- | --- | --- | --- | --- | --- |
| Yield | 3.08(5) | 3.31(1) | 2.32(9) | 3.29(2) | 3.11(4) | 2.89(7) | 2.70(8) | 3.27(3) | 3.02(6) |
| NPFP | 2.22(9) | 2.62(7) | 2.39(8) | 2.64(6) | 3.42(3) | 3.7(1) | 3.51(2) | 3.03(5) | 3.40(4) |
| aNUE | 2.31(9) | 2.36(8) | 2.64(6) | 2.39(7) | 3.27(4) | 3.81(2) | 4.13(1) | 2.80(5) | 3.28(3) |

Note: The numbers in round brackets indicate sorting.

References

Alabi,T. R., Abebe, A. T., Chigeza, G., and Fowobaje, K. R. (2022). Estimation of soybean grain yield from multispectral high-resolution UAV data with machine learning models in West Africa. Remote Sensing Applications: Society and Environment. 27,100782. <https://doi.org/10.1016/j.rsase.2022.100782.>

Bruce, W. R., Rajcan, I. and Sulik, J. (2021). Classification of soybean pubescence from multispectral aerial imagery. Plant Phenomic. 2021, 9806201. <https://doi.org/10.34133/2021/9806201>.

Di Gennaro, S.F., Toscano, P., Gatti, M., Poni, S., Berton, A. and Matese, A. (2022). Spectral comparison of UAV-based hyper and multispectral cameras for precision viticulture. Remote Sensing. 14,449. <https://doi.org/10.3390/rs14030449>.

Fei, S., Hassan, M. A., He, Z., Chen, Z., Shu, M., Wang, J., et al. (2021). Assessment of ensemble learning to predict wheat grain yield based on UAV-multispectral reflectance. Remote Sensing. 13,12,2338. <https://doi.org/10.3390/rs13122338>.

Fei, S., Hassan, M. A., Ma, Y., Shu, M., Chen, Q., Li, Z., et al. (2021). Entropy weight ensemble framework for yield prediction of winter wheat under different water stress treatments using unmanned aerial vehicle-based multispectral and thermal data. Frontiers in Plant Science. 12,730181. doi: 10.3389/fpls.2021.730181.

Fu, Z., Jiang, J., Gao, Y., Krienke, B., Wang, M., Zhong, K., et al. (2020). Wheat growth monitoring and yield estimation based on multi-rotor unmanned aerial vehicle. Remote Sensing. 12,508. <https://doi.org/10.3390/rs12030508>.

Habibullah, M., Mohebian, M. R., Soolanayakanahally, R., Bahar, A. N., Vail, S., Wahid, K. A., et al. (2020). Low-cost multispectral sensor array for determining leaf nitrogen status. Nitrogen. 1,1,67-80. <https://doi.org/10.3390/nitrogen1010007>.

Han, L., Yang, G., Dai, H., Xu, B., Yang, H., Feng, H., et al. (2019). Modeling maize above-ground biomass based on machine learning approaches using UAV remote-sensing data. Plant Methods. 15,10. <https://doi.org/10.1186/s13007-019-0394-z>.

Hassan, M. A., Yang, M., Rasheed, A., Yang, G., Reynolds, M., Xia, X., et al. (2019). A rapid monitoring of NDVI across the wheat growth cycle for grain yield prediction using a multi-spectral UAV platform. Plant Science. 282,95-103. doi: 10.1016/j.plantsci.2018.10.022.

Jay, S., Baret, F., Dutartre, D., Malatesta, G., Heno, S., Comar, A., et al. (2019). Exploiting the centimeter resolution of UAV multispectral imagery to improve remote-sensing estimates of canopy structure and biochemistry in sugar beet crops. Remote Sensing of Environment. 231. doi: 10.1016/j.rse.2018.09.011.

Jin, X., Liu, S., Baret, F., Hemerlé,M., and Comar, A. (2017). Estimates of plant density of wheat crops at emergence from very low altitude UAV imagery. Remote Sensing of Environment. 198,105-114. <https://doi.org/10.1016/j.rse.2017.06.007>.

Shafiee, S., Lied, M. L., Burud I., Dieseth, J. A., Alsheikh, M., and Lillemo, M. (2021). Sequential forward selection and support vector regression in comparison to LASSO regression for spring wheat yield prediction based on UAV imagery. Computers and Electronics in Agriculture. 183,106036.

Wan, L., Cen, H., Zhu, J., Zhang, J., Zhu, Y., Sun, D., et al. (2020). Grain yield prediction of rice using multi-temporal UAV-based RGB and multispectral images and model transfer – a case study of small farmlands in the South of China. Agricultural and Forest Meteorology. 10,8096. doi:10.1016/j.agrformet.2020.108096.

Yang, M., Hassan, M. A., Xu, K., Zheng C., Rasheed, A., Jin, X., et al, (2020). Assessment of water and nitrogen use efficiencies through UAV-based multispectral phenotyping in winter wheat. [Frontiers in Plant Science](https://www.x-mol.com/paper/journal/2185?r_detail=1276625360322588672). 11,927. doi: 10.3389/fpls.2020.00927.

Zhang, X., Zhang, K., Sun, Y., Zhao, Y., Zhuang, H., Ban, W., et al. (2022). Combining spectral and texture features of UAS-based multispectral images for maize leaf area index estimation. Remote Sensing. 14,2,331. <https://doi.org/10.3390/rs14020331>.
